# Hyperglycaemia in pregnancy: the role of ethnicity and geography in risk and outcomes

**DOI:** 10.1007/s00125-025-06510-7

**Published:** 2025-08-04

**Authors:** Lili Yuen, Wesley Hannah, Matthew Hare, David Simmons

**Affiliations:** 1 Translational Health Research Institute, Western Sydney University, Campbelltown, NSW Australia; 2 School of Medicine and Public Health, University of Newcastle, Gosford, NSW Australia; 3 Madras Diabetes Research Foundation, Chennai, India; 4 Menzies School of Health Research, Charles Darwin University, Darwin, NT Australia; 5 Endocrinology Department, Royal Darwin Hospital, Darwin, NT Australia; 6 School of Medicine, Western Sydney University, Campbelltown, NSW Australia

**Keywords:** Adverse long-term outcomes, Adverse perinatal outcomes, Diagnostic criteria, Early gestational diabetes mellitus, Epidemiology, Equity, diversity and inclusion, First trimester, Gestational diabetes mellitus (GDM), Glucose, Heterogeneity, Hyperglycaemia in pregnancy, Insulin, Late gestational diabetes mellitus, Management, Metformin, Obstetric monitoring, Pregnancy, Review, Screening

## Abstract

The global prevalence of hyperglycaemia in pregnancy (HIP) is rising alongside increases in the prevalence of obesity and diabetes. The IDF estimates that 19.7% of live births in 2024 were affected, with 79% of cases due to gestational diabetes mellitus (GDM) and 20% due to overt diabetes in pregnancy and pre-existing diabetes in pregnancy combined. HIP is linked to complications for both mother and child, including long-term health risks. Significant ethnic and geographical variations exist in the prevalence and outcomes of HIP, with women from South Asia being at the highest risk of GDM. Variations in prevalence of HIP exist both between regions (e.g. 13.8% in Africa compared with 31.8% in South-East Asia) and within individual countries. Social determinants of health, such as healthcare access and delivery, economic stability, discrimination, migration, lifestyle and other sociocultural factors, along with environmental, biological and genetic factors, contribute to these ethnic differences. Geography impacts risk through factors such as seasonality, pollution and rurality. Further variation occurs due to substantial diversity in national approaches to screening and diagnostic criteria. Ethnic disparities in GDM outcomes include variations in complications such as preeclampsia and preterm delivery. To address these disparities, a standardised and cost-effective approach to GDM screening and diagnosis that reflects the ethnic diversity in glucose profiles is recommended. Locally tailored, national prevention strategies for those with prior GDM should be introduced as a matter of urgency. Furthermore, each country should implement tailored HIP management policies and guidelines that include strategies to address the ethnic, geographical and social disparities in outcomes.

**Graphical Abstract:**

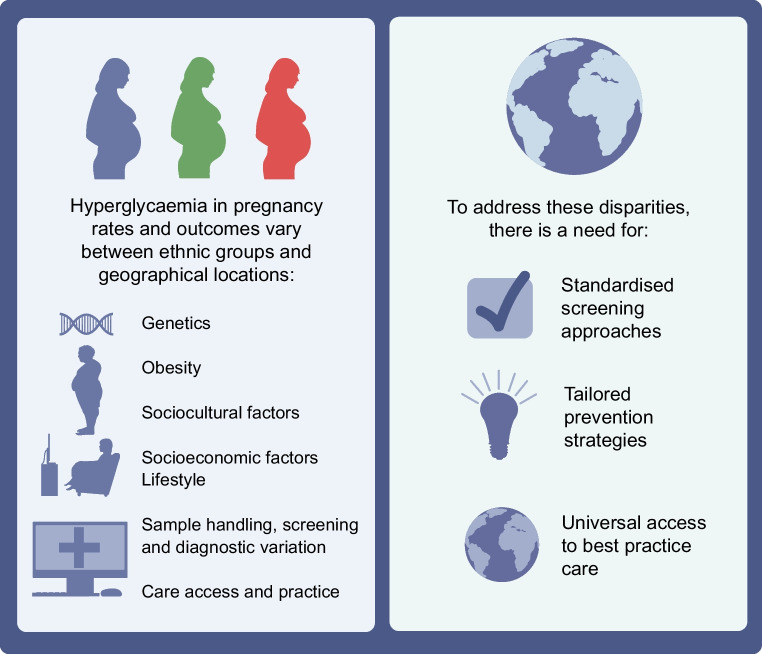

**Supplementary Information:**

The online version contains a slideset of the figures for download available at 10.1007/s00125-025-06510-7.

## Introduction

Just as the world is facing a pandemic of diabetes in non-pregnant adults, so have the numbers of women with hyperglycaemia in pregnancy been increasing. This is occurring in the context of a decades-long epidemic of diabetes and obesity across the life course, with an increasing prevalence of youth-onset type 2 diabetes [1], alongside substantial increases in the mean age at the time of conception [2]. The IDF estimated that 23.0 million (19.7%) live births were affected by hyperglycaemia in pregnancy globally in 2024 [3]. However, the age-adjusted prevalence of hyperglycaemia in pregnancy varies significantly across geographical regions, from 13.8% in Africa to 31.7% in South-East Asia (Table 1), and is influenced by environmental, genetic, sociocultural and healthcare system-related factors.

**Table 1:** Regional prevalence of hyperglycaemia in pregnancy and IGT/IFG among women aged 20–49 years

IDF region	HIP		IGT prevalence (non-pregnant) (%)	IFG prevalence (non-pregnant) (%)
	Age-adjusted prevalence (%)	Number of live births affected (millions)		
World	19.7	23.0	9.6	7.3
SEA	31.7	7.1	11.6	11.1
NAC	22.4	1.4	8.8	8.4
WP	19.8	4.2	10.7	6.5
MENA	19.4	3.6	10.1	5.9
SACA	15.8	1.0	9.9	7.7
EUR	14.2	1.5	4.0	3.6
AFR	13.8	4.7	8.7	6.0

Data for HIP reproduced from the IDF diabetes atlas, 11th edn [3] with permission. Data for non-pregnant women obtained from IDF raw data [3]

AFR, Africa; EUR, Europe; HIP, hyperglycaemia in pregnancy; MENA, Middle East and North Africa; NAC, North America and Caribbean; SACA, South and Central America; SEA, South-East Asia; WP, Western Pacific

**Fig. 1:**
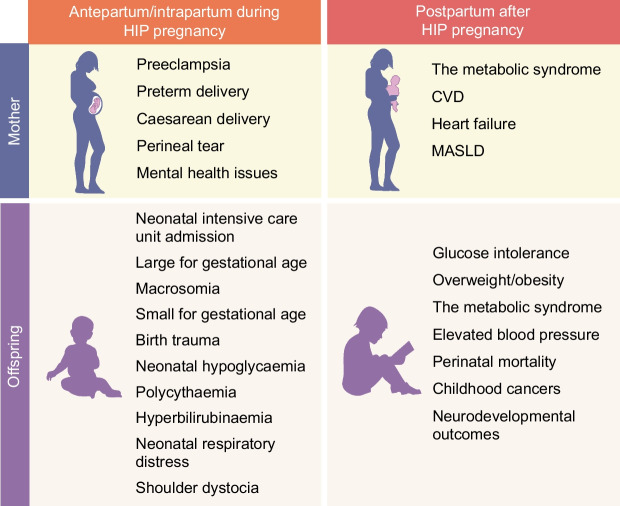
Adverse pregnancy outcomes associated with hyperglycaemia in pregnancy (HIP). MASLD, metabolic dysfunction-associated steatotic liver disease. This figure is available as part of a downloadable slideset

Hyperglycaemia in pregnancy is associated with a range of complications. However, separating the extent to which risk factors for hyperglycaemia in pregnancy, such as obesity, contribute to complications can be difficult. Figure 1 summarises the short- and long-term complications associated with hyperglycaemia in pregnancy. Lactation can also be affected [4], although few epidemiological data exist on the impact of hyperglycaemia in pregnancy on breastfeeding.

Understanding ethnic and geographical variations in the epidemiology of hyperglycaemia in pregnancy is critical to gain insights into its aetiology and pathophysiology, as well as develop region-specific strategies for diagnosis, management and prevention. This review synthesises the current evidence on ethnic and geographical variations in the prevalence and outcomes of hyperglycaemia in pregnancy.

## Defining hyperglycaemia in pregnancy

Hyperglycaemia in pregnancy encompasses pre-existing diabetes in pregnancy, gestational diabetes mellitus (GDM) and overt diabetes in pregnancy (DIP). Pre-existing diabetes in pregnancy includes known type 1 diabetes, type 2 diabetes and rarer forms of diabetes. The WHO and ADA classify pregnant women without pre-existing diabetes but fulfilling non-pregnant diabetes diagnostic criteria as having either ‘diabetes in pregnancy’ [5] or ‘diabetes complicating pregnancy’ [6] respectively. Nevertheless, permanent diabetes is often not present postpartum using these criteria [7].

Many women also enter pregnancy with either identified or unidentified impaired glucose tolerance (IGT) or impaired fasting glucose (IFG). Again, the prevalence of these forms of dysglycaemia in non-pregnant adults varies between and within geographical regions (Table 1). To address the confusion over ‘what is GDM’ created by these lesser forms of dysglycaemia (also known as ‘intermediate hyperglycaemia’ [8]), the 2013 WHO criteria define all forms of hyperglycaemia less than overt diabetes as being GDM, including previously identified or unidentified IFG, IGT or ‘prediabetes’ and hyperglycaemia fulfilling the criteria for GDM on an OGTT [5].

In this review, the 2013 WHO definitions of diabetes in pregnancy and GDM will be used, as outlined above [5]. In addition, the term ‘women’ is used in this review, as it is preferred by the majority of those with hyperglycaemia in pregnancy, although it is recognised that there are other forms of gender self-identification.

## Defining ethnicity

‘Ethnicity’ is defined in the Oxford English Dictionary as ‘membership of a group regarded as ultimately of common descent, or having a common national or cultural tradition’ [9]. Ethnicity as a term reflects the difficulty in unbundling biological, cultural and social determinant influences on health outcomes, and implies a more subjective tone with preference for classification through self-identification with a particular culture or tradition.

Race, ancestry, and ethnicity are distinct yet often overlapping concepts used to describe human diversity [10]. Race is primarily a construct that categorises people based on physical traits such as skin colour, hair texture and facial features, but it has no consistent biological basis and is shaped by, yet excludes consideration of, cultural and socioeconomic factors. Ancestry, on the other hand, refers specifically to a person’s line of descent and can be traced geographically, genealogically or genetically; it reflects the actual lineage or origins of an individual’s forebears, often identified through DNA or family history [11]. While race is imposed and often used to create social hierarchies, ethnicity is typically self-identified and rooted in cultural experience, whereas ancestry provides a factual, lineage-based perspective that may or may not align with socially defined racial or ethnic categories [12].

Defining ethnicity is increasingly difficult as global migration leads to greater degrees of admixture. Moreover, there are often clear biological, cultural and lifestyle differences, even within ethnic groups.

## Pre-existing diabetes in pregnancy

**Fig. 2:**
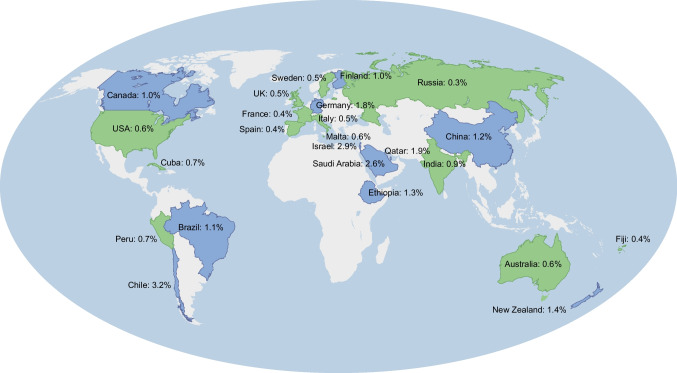
Prevalence of pre-existing diabetes in pregnancy by country from studies published since 2010. Shading represents the locations where data were available. Countries with a prevalence of pre-existing diabetes in pregnancy <1.0% are shaded in green and countries with a prevalence ≥1.0% are shaded in blue. Data from Chivese et al [16] (Australia, Canada, China, Cuba, Ethiopia, Fiji, Finland, India, Israel, Malta, New Zealand, Qatar, Saudi Arabia, Spain, Sweden, UK, USA), dos Santos et al [148] (Brazil), Garmendia et al [154] (Chile), Larrabure-Torrealva et al [155] (Peru), Postoev et al [156] (Russia), Billionnet et al [157] (France), Tamayo et al [158] (Germany) and Silvano et al [159] (Italy). This figure is available as part of a downloadable slideset

Although less common, pre-existing diabetes in pregnancy (whether diagnosed or undiagnosed) is accompanied by more serious sequelae than GDM [13–15]. Globally, pre-existing diabetes is estimated to affect approximately 0.6% of pregnancies [16], with marked variation across geographical regions (Fig. 2) and a doubling in prevalence between 1990 and 2020, mainly due to the increasing prevalence of type 2 diabetes [16]. International prevalence comparisons mask significant within-country variations. These differences largely relate to onset of type 2 diabetes at younger ages, with social and economic determinants of health considered to be key drivers [1]. Indigenous populations with a similar history of European colonisation have particularly high prevalences of pre-existing diabetes in pregnancy [17]. In the 1960s–1970s, a prevalence of pre-existing diabetes in pregnancy of 6.3% was uncovered in the Akimel O’odham (previously referred to in the literature as ‘Pima Indian’) community in Arizona [18], while a prevalence of 8.4% was reported more recently among Aboriginal women in central Australia [19].

Available estimates of the prevalence of pre-existing diabetes in pregnancy are likely to underestimate the true burden because of the high prevalence of undiagnosed diabetes, especially in low- and middle-income countries (LMICs) [20]. In addition, pre-existing diabetes is associated with increased risk of pregnancy loss and higher rates of termination due to congenital anomalies, and these pregnancies will not have been captured in studies that use total births or live births as the denominator [21].

Multiple studies have demonstrated disparities in pregnancy outcomes between women with pre-existing diabetes of different ethnic backgrounds who live in the same geographical locations [22–24]. Non-White minority groups in countries with predominantly populations of European descent (hereafter referred to as ‘European’), including the UK and USA, have an increased risk of adverse pregnancy outcomes, irrespective of type of pre-existing diabetes [22–24]. Such differences may relate to socioeconomic disparities and inequities in access to appropriate care.

While type 1 and type 2 diabetes account for the vast majority of cases of pre-existing diabetes in pregnancy, it is important to recognise other diabetes subtypes, particularly monogenic diabetes, which is caused by a mutation in a single gene, with more than 20 genes identified to date [25]. In European populations, monogenic diabetes is estimated to account for up to 3% of diabetes cases diagnosed in childhood [26]. Because of under-recognition and limited access to screening, most cases are thought to be misdiagnosed [27]. In an Irish cohort, 1% of women diagnosed with GDM were found to have monogenic diabetes caused by a variant in the *GCK* gene [28]. Other variants with not insignificant frequency that can be present at the time of conception have been found in specific populations, including the Akimel O’odham people [29], Inuit people in Greenland [30] and Oji-Cree people in Canada [31]. Among Samoan people, a frequent missense variant has been associated with an increased risk of obesity but lower risk of type 2 diabetes [32].

## Newly identified diabetes in pregnancy

Ethnic differences in the prevalence of newly identified DIP will depend on the prevalence of pre-existing diabetes in pregnancy (Fig. 2) and the penetration of screening activities (including postpartum in women with prior GDM). The prevalence of DIP in one multiethnic cohort was 0.8% overall [33]. In a multiethnic population in Australia, 14.8% of women with newly identified hyperglycaemia in pregnancy had DIP, with a rate of 9.4% among European women but rates of 23.9% in South Asian, 16.4% in Middle Eastern, 11.3% in South-East Asian and 23.1% in other (Māori, Pacific Islander, Samoan, non-White African) women [34]. In a Pakistani cohort, 16.8% had DIP and only 2% had pre-existing DIP [35]. While DIP is often associated with worse pregnancy outcomes [36–38], ethnic differences have not been studied and are likely to mirror ethnic differences in outcomes associated with known pre-existing diabetes in pregnancy.

## Gestational diabetes mellitus

### Diagnosis

Diagnostic approaches for GDM vary around the world, contributing to the observed differences in prevalence. Originally, the 100 g 3 h OGTT was devised to diagnose GDM based on the prediction of maternal glucose intolerance. At least two abnormal values after the glucose load (defined as two SDs above the mean glucose level at 0, 1, 2 and/or 3 h) were to be met or exceeded, to avoid laboratory error or single peaks from the rapid absorption of glucose [39]. Over the last few decades, the US National Diabetes Data Group (NDDG) and the Carpenter and Coustan (C&C) GDM criteria [40] (Table 2) refined these original criteria to account for technological advances in the way that glucose is measured [39, 41–43]. There remains a large variety of methods in use across geographical locations as shown in Table 2 and, in the USA, both NDDG and C&C criteria are used.

**Table 2:** Approaches to diagnosing GDM

GDM diagnostic test or professional body	Year	Screening approach	OGTT used	Diagnostic criteria	Screening time period	Early diagnosis?
International Association of Diabetes and Pregnancy Study Groups (IADPSG) [46]	2010	Universal one-step approach	75 g	IADPSG 2010 criteria [46] for the diagnosis of GDM Perform a single 75 g OGTT; diagnosis of GDM is made when one or more of the following plasma glucose levels are met: • FPG: ≥5.1 mmol/l • 1 h post-challenge glucose level: ≥10.0 mmol/l • 2 h post-challenge glucose level: ≥8.5 mmol/l	24–28 weeks	If high risk (defined by local criteria) tested at first prenatal visit; if negative, retested along with general population at 24–28 weeks’ gestation
WHO [5]	2013	Universal one-step approach	75 g	IADPSG 2010 criteria [46]	24–28 weeks	As soon as feasible for those at high risk; retested if negative at 24–28 weeks’ gestation
International Federation of Gynaecology and Obstetrics (FIGO) [42]	2015	Universal one-step approach	75 g	WHO 2013 [5] and IADPSG 2010 [46] criteria	24–28 weeks	• Fully resourced settings: FPG, RBG or HbA_1c_ to detect DIP • Fully resourced settings serving high-risk ethnic groups: 75 g 2 h OGTT to detect DIP
ICD codes		Coding		ICD-11 codes: JB63.0 (Pre-existing type 1 DIP), JB63.1 (Pre-existing type 2 DIP), JB63.2 (GDM), JB63.Y (Other specified DIP), JB63Z (Unspecified DIP) ICD-10 codes: O24.0 (Pre-existing type 1 DIP), O24.1 (Pre-existing type 2 DIP), O24.2 (Pre-existing malnutrition-related DIP), O24.3 (Pre-existing DIP unspecified), O24.4 (GDM), O24.9 (DIP unspecified) ICD-9 codes: 648.0x (DIP), 648.8x (GDM)		
ADA [6]/American College of Obstetricians and Gynecologists (ACOG) [122]	2014/2018	Universal one-step approach or universal two-step approach (C&C)		IADPSG 2010 [46] or C&C [40] Non-fasting 50 g GCT followed by a 3 h 100 g OGTT when the 1 h measurement is ≥7.5 mmol/l GDM if two or more of: • FPG ≥5.3 mmol/l • 1 h PG ≥10.0 mmol/l • 2 h PG ≥8.6 mmol/l • 3 h PG ≥7.8 mmol/l Diabetes complicating pregnancy if: • FPG ≥7.0 mmol/l and/or • 2 h PG ≥11.1 mmol/l	24–28 weeks	Presence of risk factors 1−13^a^ or Black, Hispanic, Native American, Asian American or Pacific Islander origin^b^ Early abnormal glucose metabolism if FPG 6.1–6.9 mmol/l or HbA_1c_ 41–46 mmol/mol (5.9–6.4%)
NDDG [43] USA	1979	Universal two-step approach	100 g	Non-fasting 50 g GCT ≥7.8 mmol/l followed by a 3 h 100 g OGTT if positive GDM if two or more of: • FPG ≥5.8 mmol/l • 1 h PG ≥10.6 mmol/l • 2 h PG ≥9.2 mmol/l • 3 h PG ≥8.0 mmol/l	24–28 weeks	
O’Sullivan and Mahan [39] USA (no longer in use)	1964	Universal one-step approach using whole blood (Somogyi–Nelson method) rather than plasma	100 g	GDM if two or more of: • FPG ≥5.0 mmol/l • 1 h PG ≥9.2 mmol/l • 2 h PG ≥8.0 mmol/l • 3 h PG ≥6.9 mmol/l	24–28 weeks	
Australasian Diabetes in Pregnancy Society (ADIPS) [123]	2014	Universal one-step approach	75 g	IADPSG 2010 criteria [46]	24–28 weeks	Presence of risk factors 2, 6, 9, 10, 14−17^a^ plus Asian, Indian subcontinent, Aboriginal, Torres Strait Islander, Pacific Islander, Mãori, Middle Eastern and non-White African individuals^b^
Australian Diabetes in Pregnancy Society (ADIPS) (updated [124])	2025	Universal one-step	75g	GDM if one or more of: • FPG ≥5.3 mmol/l • 1 h PG ≥10.6 mmol/l • 2 h PG ≥9.0 mmol/l	24–28 weeks	Presence of risk factors 1, 2, 6, 10, 16, 18–22^a^ before 20 weeks, test with first-trimester HbA_1c_: if ≥48 mmol/mol (6.5%), treat as overt DIP; if between 42 mmol/mol (6.0%) and 46 mmol/mol (6.4%) or previous GDM perform OGTT (local policy may allow diagnosis of GDM without OGTT) Also allows for broader local early screening policy, e.g. using the risk factor approach for both early HbA_1c_ and early OGTT
Chinese Ministry of Health [125]	2011	Universal one-step screening approach in high-resourced settings and two-step approach in low-resourced settings	75 g	IADPSG 2010 criteria [46] Low-resourced settings: FPG used as a screening tool to reduce number of OGTTs required. If FPG ≥5.1 mmol/l, GDM is diagnosed; if <4.4 mmol/l, GDM unlikely. Between 4.4 and 5.0 mmol/l, OGTT required	24–28 weeks	FPG at first visit. If ≥5.6 mmol/l, diagnosed with IFG
Danish Society of Obstetrics and Gynaecology [126] (under review)	2014	Universal one-step approach	75 g	GDM if: • FPG ≥5.3 mmol/l and/or • 2 h PG ≥9.0 mmol/l	28 weeks	Screening at 18 gestational weeks if more than one of the following risk factors is present: 2, 10, 16, 23, 24^a^
Diabetes Canada Clinical Practice Guidelines [127]/Society of Obstetricians and Gynaecologists of Canada [128]	2018/2019	Universal screening via a 50 g GCT (preferred) or universal screening via a 75 g OGTT	75 g	Preferred approach: screening via a 50 g GCT. If 1 h PG is ≥11.1 mmol/l, GDM is diagnosed. If 1 h PG value is between 7.8 and 11.0 mmol/l, a 2 h 75 g OGTT is conducted. GDM if one of FPG ≥5.3 mmol/l, 1 h PG ≥10.6 mmol/l or 2 h PG ≥9.0 mmol/l Alternative approach: IADPSG 2010 criteria [46]	24–28 weeks	If high risk of GDM based on multiple clinical factors, screening should be offered at any stage in pregnancy
Deutsche Gesellschaft für Gynäkologie und Geburtshilfe (German Society of Gynecology and Obstetrics) [129]	2018	Universal one-step approach	75 g	IADPSG 2010 criteria [46] If FPG with hand-held device ≥6.9 mmol/l and no OGTT, diagnosis of DIP can be made	24–28 weeks	Women with increased risk should be screened before 24 weeks’ gestation; GDM diagnosed in those with two FPG ≥5.1 mmol/l on different days, or HbA_1c_ >41 mmol/mol (5.9%) and OGTT or FPG ≥5.1 mmol/l Increased risk is based on risk factors 2, 10, 11, 16, 25, 26^a^ or East Asian or South Asian origin^b^
Diabetes in Pregnancy Study Group India (DIPSI) [130, 131]	2014	Universal non-fasting OGTT	75 g	GDM if 2 h PG ≥7.8 mmol/l; diabetes if 2 h PG ≥11.1 mmol/l	24–28 weeks	Universal screening performed at the first antenatal visit and then, if negative, repeated at 24–28 weeks
Finnish Medical Society [132]	2008	Universal one-step approach except for those at low risk	75 g	GDM if one of: • FPG ≥5.3 mmol/l • 1 h PG ≥10.0 mmol/l • 2 h PG ≥8.6 mmol/l	24–28 weeks	12–16 weeks’ gestation if the following risk factors are present: 6, 10, 17, 19, 24^a^
Flemish consensus on screening for GDM [133] Belgium	2024	Universal one-step approach	75 g	IADPSG 2010 criteria [46]	24–28 weeks	Before 20 weeks’ gestation: 75 g OGTT if risk factors 10 or 15 (≥30 kg/m^2^)^a^ GDM if any of: • FPG ≥5.3 mmol/l • 1 h PG ≥10.6 mmol/l • 2 h PG ≥9.0 mmol/l
Grupo Español de Diabetes y Embarazo (Spanish Diabetes and Pregnancy Group; GEDE) [134]	2021	Two-step approach	100 g	NDDG criteria [43]	24–28 weeks	Screening at 10–12 weeks’ gestation for high-risk women: risk factors 2, 10, 11, 15, 16 or 18^a^ or Latin American or South-East Asian individuals^b^
Irish Health Services Executive (HSE) [135]	2010	Selective screening; risk factor-based	75 g	IADPSG 2010 criteria [46]	24–28 weeks if risk factors 2, 6, 14–18, 24 or 27^a^ are present or belonging to an ethnic group with a high prevalence of diabetes	
Japan Society of Obstetrics and Gynecology (JSOG) and Japan Association of Obstetricians and Gynecologists (JAOG) [136]	2011	Universal two-step approach	75 g	All women undergo a 50 g GCT; if ≥7.8 mmol/l at 1 h, women undergo a 75 g OGTT, with diagnosis based on IADPSG 2010 criteria [46]	24–28 weeks	All women with RBG ≥5.8 mmol/l undergo a 75 g OGTT during the first trimester
National Institute for Health and Care Excellence (NICE) [49, 137] UK	2008; 2015	Risk-based assessment	75 g	GDM if: • FPG ≥5.6 mmol/l and/or • 2 h PG ≥7.8 mmol/l	24–28 weeks if risk factors 2, 10, 15 or 16^a^ are present or belonging to a minority ethnic group with a high prevalence of diabetes^b^	For those with previous GDM, early self-monitoring of blood glucose; otherwise, OGTT at booking (first antenatal clinic attendance)
New Zealand Ministry of Health (NZMOH) [138]	2014	Universal two-step		For women whose booking HbA_1c_ was 41–49 mmol/mol (5.9–6.6%), GDM if fasting glucose is ≥5.5 mmol/l or 2 h value is ≥9.0 mmol/l For women whose booking HbA_1c_ was ≤40 mmol/mol (5.8%), perform 50 g GCT: GDM if glucose is ≥11.1 mmol/l, or if glucose is ≥7.8–11.0mmol/l, perform a 2 h OGTT with above cut-off	24–28 weeks	Routine HbA_1c_ before 20 weeks’ gestation: if ≥50 mmol/mol (≥6.7%) has HIP; if HbA_1c_ 41-49 mmol/mol (5.9–6.6%) undergo OGTT at 24-28 weeks
Swedish National Board of Health and Welfare [139]	2004; 2015	Selective	75 g	IADPSG 2010 criteria [46] set nationally (2015) Many regions still use 2004 criteria (diagnosed via either serum or capillary plasma glucose levels): GDM if: • FPG ≥7.0 mmol/l and/or • 2 h PG ≥10.0 mmol/l Criteria also vary by region, with FPG used or not, and 2 h PG cut-off ranging from 9.0 to 11.1 mmol/l	24–28 weeks Variable risk factors across Sweden or RBG ≥9.0 mmol/l	If RBG ≥9.0 mmol/l perform OGTT

^a^Risk factors: (1) Overweight or obesity (pre-pregnancy BMI ≥25 kg/m^2^ overall or ≥23 kg/m^2^ for Asian individuals); (2) first-degree relative with diabetes; (3) history of CVD; (4) hypertension (i.e. ≥140/90 mmHg or on therapy for hypertension); (5) previous history of hyperlipidaemia (i.e. HDL-cholesterol level <0.90 mmol/l, triglyceride level >2.82 mmol/l); (6) previous history of polycystic ovary syndrome; (7) physical inactivity; (8) other clinical conditions associated with insulin resistance (e.g. severe obesity, acanthosis nigricans); (9) prediabetes (i.e. HbA_1c_ ≥39 mmol/mol [5.7%], impaired glucose tolerance or impaired fasting glucose); (10) previous GDM diagnosis; (11) maternal age ≥35 years; (12) previous history of HIV/AIDS; (13) other factors suggestive of an increased risk for pregestational diabetes; (14) maternal age ≥40 years; (15) pre-pregnancy BMI >30 kg/m^2^; (16) previous history of macrosomia (baby with birthweight >4500 g or >90th centile); (17) taking corticosteroids or antipsychotics; (18) previous history of polyhydramnios; (19) pre-pregnancy BMI ≥35 kg/m^2^; (20) hypothyroidism; (21) maternal age >30 years; (22) previous adverse outcomes including preterm delivery, stillbirth and congenital abnormality and pre-eclampsia; (23) overweight (pre-pregnancy BMI ≥27 kg/m^2^); (24) previous history of glycosuria; (25) weight >69 kg; (26) height <1.64 m; (27) previous unexplained perinatal death

^b^Descriptions of ethnic groups are as used in the original publications

FPG, fasting plasma glucose; HIP, hyperglyaemia in pregnancy; PG, plasma glucose; RBG, random blood glucose

Outside pregnancy, a 75 g 2 h OGTT is used to diagnose diabetes, and this has been adopted as the antenatal test across many jurisdictions instead of the 100 g 3 h OGTT (Table 2), not only to standardise the approach across the life course, but also because of the time-consuming, unpleasant and more side effect-prone nature of the 100 g 3 h OGTT [44]. In 2010, the International Association of Diabetes in Pregnancy Study Groups (IADPSG) used 75 g 2 h OGTT data at 24–32 weeks’ gestation from the Hyperglycaemia And Pregnancy Outcomes (HAPO) study (*n*=23,316 women from 15 centres in nine countries) [45] to define GDM diagnostic thresholds based on three neonatal outcomes (birthweight >90th percentile, cord C-peptide levels >90th percentile, and neonatal per cent body fat >90th percentile) [46] (see Text box: IADPSG/WHO criteria for the diagnosis of GDM). The IADPSG criteria require only one abnormal value but have not been universally accepted as shown in Table 2. Some of the variation in GDM diagnostic approaches occurs because of lack of resources [47], ethnic differences in fasting glycaemic risk [48] and difficulties in attending for a fasting blood glucose test (e.g. in India [47]).


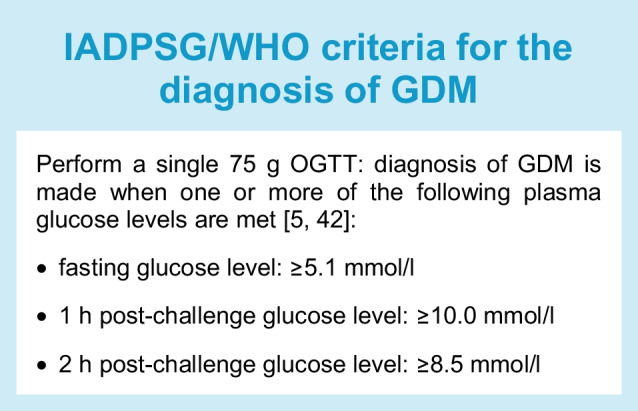


HbA_1c_ measurement has not been found to be useful for the diagnosis of GDM because of its low sensitivity [49–51]; in addition, results can vary by, for example, ethnic variation in red cell turnover, including risk of anaemia [52].

### Screening

Whether to perform an OGTT in all women (universal screening) or only in those considered at highest risk (selective screening) is debated based on considerations of clinical effectiveness, resource allocation and the potential impact on the pregnancy experience. Table 2 lists the risk factors considered in different approaches. Ethnicity is included as a risk factor in selective screening approaches in most jurisdictions with majority European populations (Table 2).

Among those referred for screening, either a one-step (straight to OGTT) or a two-step (OGTT only if a prior blood test is positive) approach can be used. The IADPSG and WHO recommend a one-step approach for simplicity, whereas the USA use the NDDG and C&C classifications, which involve a two-step approach that uses a non-fasting 1 h 50 g glucose challenge test (GCT) (with various thresholds from 7.2 to 7.8 mmol/l) followed by a 3 h 100 g OGTT if positive [53–55]. The GCT is generally only 85% sensitive with markedly less sensitivity among ethnic groups whose GDM is associated more with fasting hyperglycaemia [56], but can reduce the total number of OGTTs performed overall. However, using this approach disproportionately exposes women from ethnic groups with a higher risk of GDM, who are more likely to test positive on the GCT (e.g. rural Australia: GCT positivity 28.6% vs 12.5% in Aboriginal vs non-Aboriginal women [57]), to increased side effects from use of a 3 h OGTT, which has a three- to fourfold increased risk of side effects compared with the 75 g OGTT.

**Fig. 3:**
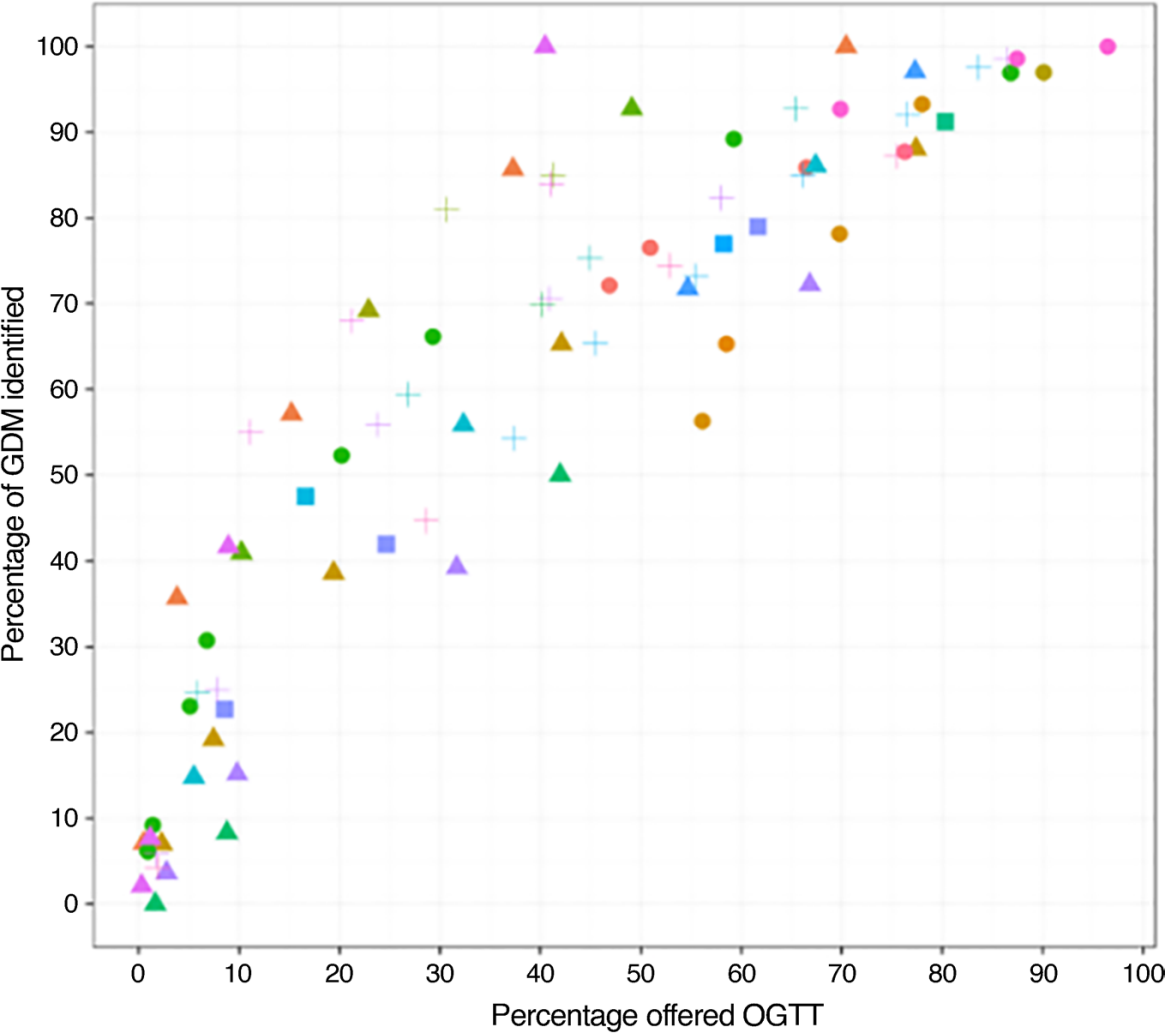
Sensitivity of different screening approaches for identifying GDM, illustrating that detection of GDM in a population is largely proportionate to the number of OGTTs undertaken. Individual points represent single studies, with circles representing studies using guideline-based screening (e.g. group of risk factors or 50 g GCT), triangles representing studies using risk factor screening, squares representing studies using other types of screening and crosses representing studies using risk models/score-based screening. These different approaches to screening triage different proportions to being offered an OGTT (x axis). The y axis shows the proportion of women with GDM identified (sensitivity). Reproduced from Farrar et al [58] under the terms of the CC BY 4.0 Attribution License (http://creativecommons.org/licenses/by/4.0/); for details of individual studies see Farrar et al [58]. This figure is available as part of a downloadable slideset

GDM screening methods other than the one-step universal approach all reduce sensitivity and therefore the effectiveness of population-based GDM management. A meta-analysis across multiple populations showed that the proportion of GDM detected correlated with the proportion of women offered an OGTT, whether based on the presence of risk factors or other guidelines (Fig. 3) [58].

### Glucose measurement

One of the failings of the OGTT is that it has limited replicability and reliability [41, 59]. The original conversion of whole blood glucose to plasma glucose thresholds according to the O’Sullivan method (NDDG [43] and C&C [40]) may have had a differential impact on reported prevalence of GDM and validity across ethnic groups because it did not account for population differences in haematocrit values relating to the prevalence of anaemia [60]. In addition, currently, most of the glucose variation arises from pre-analytical issues, including within-person day-to-day variation [61]. OGTTs have been shown to underestimate GDM prevalence in remote Australia [62] due to pre-analytical glycolysis, which can be mitigated by rapid plasma separation or use of citrate tubes [41]. In one hospital service, switching from delayed to early centrifugation of blood samples led to an increase in GDM diagnosis rate from 11.6% to 20.6% [63].

### Early GDM

GDM has traditionally been diagnosed between 24 and 28 weeks’ gestation, reflecting the more severe insulin resistance at this time, which exposes those with reduced insulin secretory capacity to relative insulinopenia and resulting hyperglycaemia. Treatment for GDM is beneficial from this time [64]. However, the growing numbers of women with pre-pregnancy IGT and IFG, and their identification during early screening for DIP, has led to recognition of the need to consider such women for treatment [65]. Pregnancy complications are associated with hyperglycaemia in early pregnancy and correlate linearly with early glucose levels [66], and women in whom GDM is identified early are at greater risk of adverse pregnancy outcomes [67]. First-trimester GDM screening using an OGTT among women with obesity is associated with reductions in large-for-gestational-age (LGA) births (adjusted OR [aOR] 0.89 [95% CI 0.82, 0.96]) and Caesarean deliveries (0.89 [95% CI 0.85, 0.93]) across the obstetric population, with even greater reductions among women with GDM (0.52 [95% CI 0.39, 0.70] and 0.78 [95% CI 0.65, 0.94] respectively) [68]. A large RCT of treatment of early GDM (defined as GDM present before 20 weeks’ gestation) in a multiethnic cohort showed reductions in neonatal respiratory distress and neonatal intensive care unit length of stay [33, 69]. No ethnic differences in outcomes were found [70]. With the recognition that treating early GDM is associated with improved pregnancy outcomes, health systems that test for GDM only after 24 weeks’ gestation will provide epidemiological data that is a conflation of both early GDM and those who developed GDM later in pregnancy [71]. Few epidemiological data exist for those who develop GDM later in pregnancy. In one study, South Asian women had the highest incidence of GDM developing de novo after early GDM had been excluded [70].

## Global epidemiology of GDM

Describing the global epidemiology of GDM is challenging, as the prevalence of GDM varies significantly depending on the screening and diagnostic approaches used (Table 2). The global standardised IADPSG-defined GDM prevalence in 2021 was 14.2% [72], varying from 6.6% in Nepal to 45.3% in the United Arab Emirates; use of the IADPSG criteria resulted in a 1.03- to 3.78-fold increase in prevalence compared with criteria in use prior to the change (e.g. WHO 1999 [73], NDDG [43], C&C [40] or multiple other national criteria) [74]. There has been a real increase in the prevalence of GDM since the 1990s, including in Australia [75] and the USA [76, 77].

## Role of ethnicity in risk and outcomes

### Ethnic differences in prevalence of GDM

**Fig. 4:**
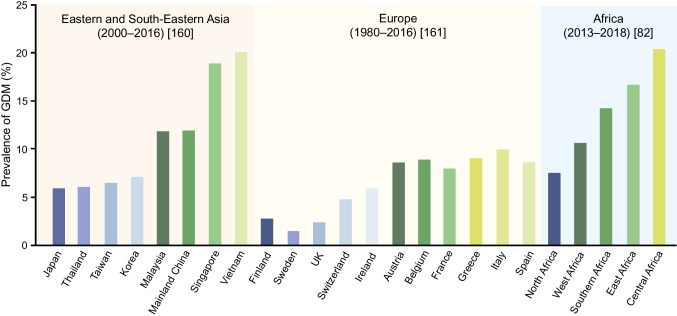
Prevalence of GDM by country. Lower prevalence (below the regional mean) countries are shown by the blue bars and higher prevalence countries are shown by the green bars. This figure is available as part of a downloadable slideset

Ethnicity has long been classified as a risk factor for GDM [78], with Green et al reporting in 1990 that there was a higher risk among Chinese women and lower risk among Black women compared with Hispanic or non-Hispanic White women [79]. Ethnicity was the dominant influence on prevalence of GDM in a UK study in 1992 [80]. Women from ethnic groups other than White had a higher frequency of GDM than White women, with adjusted RRs (95% CIs) of 3.1 (1.8, 5.5) among Black women, 7.6 (4.1, 14.1) among South-East Asian women and 11.3 (6.8, 18.8) among Indian women. Universal screening of at-risk ethnic groups was recommended in 1998 [81] and has been adopted in many guidelines (Table 2). Table 3 summarises GDM prevalence by country and ethnic group and Fig. 4 by country and region. Indigenous women consistently have a higher prevalence and European women a lower prevalence of GDM. The IDF Atlas estimated that African women have the lowest prevalence of hyperglycaemia in pregnancy at 13.8% [3], although one meta-analysis suggested higher GDM prevalences in East and Central African populations [82]. Migration may be a risk factor for GDM in some ethnic groups [83], with one meta-analysis reporting that foreign-born Hispanic women who have lived in the USA for <10 years have a lower risk for GDM (aOR 0.54, 95% CI 0.32, 0.91) than US-born Hispanic women, but foreign-born non-Hispanic Black women who have lived in the USA for <10 years have a higher risk for GDM (aOR 1.60, 95% CI 0.99, 2.60) than US-born non-Hispanic Black women.

**Table 3:** Studies reporting the prevalence of GDM by country

Study details	Screening/diagnostic method	Overall GDM prevalence	Prevalence in lower risk populations by ethnic group^a^	Prevalence in higher risk populations by ethnic group^a^
USA, 2006, 2016, 2017 *N*=37,357 [140]	Self-reported in a population-based National Health Interview Survey	8.2 (95% CI 7.5, 8.9)^b^	Non-Hispanic White 7.6 (6.7, 8.5)^b^ Non-Hispanic Black 7.3 (5.8, 8.8)^b^	Hispanic 9.5 (7.4, 11.6)^b^ Other race/ethnicity 11.1 (8.5, 13.8)^b^
USA, 2016–2022 *N*=197,236 [141]	Self-reported data from the Pregnancy Risk Assessment Monitoring System	10.7%	White 9.14% Black 8.87%	Hispanic 11.93% Chinese 17.20% Filipino 19.56% Japanese 10.80% Other Asian 18.49% Native Hawaiian 10.71%
USA, 2015–2021 *N*=344,842 [142]	ICD-9-CM or ICD-10-CM codes Live births		White 10.85% Black 9.78%	Hispanic 13.65% Other ethnicity 16.16%
Australia (19 studies), Canada (8 studies), New Zealand (1 study) and the USA (15 studies), inception to 2019 *N*=32,952,441 [17]	Systematic review and meta-analysis Data derived from meta-analysis tables overall, various criteria over time		Non-Indigenous: Australia 4.5% Canada 3.8% USA 4.7% New Zealand 2.5%	Indigenous:^c^ Australia 5.3% (1.42 [1.24, 1.63]) Canada 6.5% (2.04 [1.46, 2.84]) USA 6.9% (1.49 [1.32, 1.67]) New Zealand Mãori 5.7% (no 95% CI) NB Pasǐfika (non-Indigenous) 6.6%
UK, 2007–2011 *N*=9509 [143]	Review of individual patient data from the Born in Bradford cohort and Warwick/Coventry hospitals		White: Born in Bradford 4.9% Warwick/Coventry 8.1%	South Asian: Born in Bradford 10.8% Warwick/Coventry 10.8% Other: Born in Bradford 8.7% Warwick/Coventry 8.9%
France, 2002–2010 *N*=20,653 [144]	Single-centre study of consecutive singleton births Universal screening: GDM defined as FPG ≥5.3 mmol/l and/or 2 h home blood glucose >7.8 mmol/l	14.6%	European 12.2% Sub-Saharan African 10.9% Caribbean 13.1%	North African 17.6% South Asian 28.9% Asian 16.0%
Norway, 2008–2010 *N*=823 [145]	Population-based cohort study in three public child health clinics in Oslo Point-of-care testing	WHO 1999 criteria 13.0% IADPSG criteria 31.5%	Western European: WHO 10.9% IADPSG 24.0%	Minority ethnic groups: WHO 14.6% IADPSG 36.8%
Canada, 2004–2010 *N*=498,013 [146]	Administrative data from two provinces: British Columbia (BC) and Alberta (AB) All deliveries	AB 4.8% BC 7.2%	General population: AB 4.2% BC 5.8%	Chinese: AB 11% BC 13.5% South Asian: AB 8.4% BC 13.9%
Israel, 2007–2017 *N*=9875 [147]	District health services in one city All women undergoing OGTT		Jewish ethnicity 10.6%	Arab ethnicity 17.3%
Brazil, 2016 *N*=2313 [148]	Users of the Brazilian Unified Health System Attended prenatal follow-up visit in one city	5.4%	European descent 5.2%	African descent 10.9%

^a^Lower risk is defined as below the mean prevalence across the whole population, and higher risk is defined as above the mean prevalence across the whole population. Descriptions of ethnic groups are as used in the original publications

^b^Prevalence per 100 people

^c^aOR (95% CI)

FPG, fasting plasma glucose

### Ethnic differences in GDM outcomes

Disparities in GDM outcomes between ethnic groups have been reported, including from large databases of women with GDM living in Western countries [84]. Table 4 summarises within-country differences in maternal and neonatal outcomes by ethnicity in large GDM cohorts; although there is heterogeneity between studies in the methodology used, all of the studies included highlighted significant ethnic differences in GDM outcomes.

**Table 4:** Large studies reporting GDM adverse pregnancy outcomes that were significantly different between ethnic groups

Study details	Diagnostic method	Total GDM population	Comparative ethnicities	Complication incidence in lower risk or reference populations^a^	Complication incidence in higher risk or non-reference populations^a^
Australia 2010–2013 [149]	ADIPS 1999	1579	European 1181 Chinese 398	European: • Macrosomia (>4.0 kg): 14.3% • LGA: 18.0% • Neonatal hypoglycaemia: 11.0% • Preterm birth: 11.5% • Neonatal death: 0.6% • Stillbirth: 0.6% • Jaundice: 8.6% • Respiratory distress: 5.5% • NICU admission: 29.8%	Chinese: • Macrosomia (>4.0 kg): 5.8% • LGA: 6.5% • Neonatal hypoglycaemia: 4.5% • Preterm birth: 6.3% • Neonatal death: 0% • Stillbirth: 0% • Jaundice: 4.8% • Respiratory distress: 2.0% • NICU admission: 11.1%
New Zealand 2011–2017 [150]	NZ MOH 2014	2639 (including 573 ODIP and 493 T2D)	European 294 Pacific 952 New Zealand Mãori 324 Asian 1009 Other 60	European: • Stillbirth: 0% • Preeclampsia: 8.1% • LGA: 10.3% • SGA: 9.6%	Pacific: • Stillbirth: 1.1% • Preeclampsia: 13.5% • LGA: 30.1% • SGA: 8.0% Mãori: • Stillbirth: 0.9% • Preeclampsia: 14.0% • LGA: 22.7% • SGA: 12.9% Asian: • Stillbirth: 0% • Preeclampsia: 6.9% • LGA: 11.7% • SGA: 10.9% Other: • Stillbirth: 0% • Preeclampsia: 5.1% • LGA: 8.9% • SGA: 17.9%
Spain 1986–2007 [151]	NDDG	2543	European 2480 Non-European 63	European: • Macrosomia: 4.3%; OR 1.0 • LGA: 9.5%; OR 1.0	Non-European: • Macrosomia: 19.4%; OR 2.994 (95% CI 1.152, 7.779) • LGA: 32.3%; OR 2.767 (95% CI: 1.257, 6.091)
UK 2007–2011 [152]	ICD-10 codes	10,868 (4811 with disclosed ethnicity)	White 3318 Black 235 South Asian 777 Mixed 70 Other 411	White (HR [95% CI]): • Subsequent T2D: 1.96 (1.76, 2.18) • Hypertension: 0.82 (0.7, 0.97) • Depression: 4.67 (4.36, 5.01)	Black (HR [95% CI]): • Subsequent T2D: 2.47 (1.7, 3.57) • Hypertension: 2.29 (1.56, 3.37) South Asian: • Subsequent T2D: 3.06 (2.55, 3.68) • Depression: 1.97 (1.57, 2.48) Mixed: • Depression: 2.83 (1.42, 5.86) Other: • Depression: 2.60 (1.99, 3.4)
USA 2014-2020 [153]	As per National Center for Health Statistics protocol	1,560,822	American Indian 19,261 Asian/Pacific Islander 195,982 Black 185,629 Hispanic/Latina 415,477 White 744,473	White	American Indian (ARR [95% CI]): • Caesarean delivery: 0.98 (0.96, 0.99) • Preeclampsia or gestational hypertension: 1.24 (1.2, 1.29) • Maternal ICU admission: 1.39 (1.04, 1.85) • Tranfusion: 2.46 (2.13, 2.84) • Preterm birth: 1.10 (1.06, 1.15) • LGA: 1.24 (1.21, 1.27) • NICU admission: 1.05 (1.01, 1.09) • Macrosomia: 1.32 (1.28, 1.37) • SGA: 0.92 (0.87, 0.98) Asian/Pacific Islander: • Caesarean delivery: 0.93 (0.92, 0.94) • Preeclampsia or gestational hypertension: 0.58 (0.57, 0.59) • LGA: 0.52 (0.51, 0.53) • Macrosomia: 0.51 (0.50, 0.52) • Preterm birth: 1.02 (1.01, 1.04) • NICU admission: 1.04 (1.03, 1.06) • SGA: 1.84 (1.82, 1.87) Black: • LGA: 0.84 (0.83, 0.85) • Macrosomia: 0.78 (0.77, 0.80) • Caesarean delivery: 1.13 (1.12, 1.14) • Preeclampsia or gestational hypertension: 1.22 (1.20, 1.23) • Maternal ICU admission: 1.58 (1.43, 1.75) • Transfusion: 1.17 (1.09, 1.26) • Preterm birth: 1.40 (1.39, 1.42) • NICU admission: 1.44 (1.42, 1.46) • SGA: 1.6 (1.57, 1.62) Hispanic/Latina: • Preeclampsia or gestational hypertension: 0.88 (0.87, 0.89) • LGA: 0.95 (0.94, 0.96) • Macrosomia: 0.92 ( 0.91, 0.93) • Caesarean delivery: 1.02 (1.01, 1.02) • Maternal ICU admission: 1.19 (1.09, 1.31) • Preterm birth: 1.18 (1.17, 1.19) • NICU admission: 1.12 (1.11, 1.13)

^a^Descriptions of ethnic groups are as used in the original publications

ADIPS, Australasian Diabetes in Pregnancy Society; ARR, adjusted RR; ICU, intensive care unit; IR, incidence ratio; NA, not applicable; NICU, neonatal intensive care unit; NZ MOH, New Zealand Ministry of Health; ODIP, overt diabetes in pregnancy ; SGA, small for gestational age; T2D, type 2 diabetes

### Social, lifestyle and biological determinants of ethnic differences in GDM

By definition, ethnicity encompasses social, lifestyle and biological determinants of GDM, making it difficult to tease out the different contributing factors. Five key social determinants of health (SDOH) that impact on health and well-being have been flagged by WHO: economic stability, education, healthcare access and quality, neighbourhood and built environment, and social and community context [85, 86]. Groups at high risk of GDM, particularly Indigenous and other minority populations in high-income countries, are vulnerable to the inequitable impact of SDOH on their pregnancies and offspring [87, 88]. However, some ethnic groups with adverse SDOH have a similar or lower prevalence of GDM than White populations (e.g. non-Hispanic Black women; Table 3). Discrimination also plays a part in explaining some of the ethnic differences in GDM outcomes, as concluded by a meta-analysis of 24 predominantly USA-based studies [84, 89]. Ethnic differences in GDM screening uptake and in clinical outcomes within the same geographical location may relate to inequitable healthcare access due to factors including socioeconomic disparities and structural racism [90].

In high-income countries, standardised screening protocols and advanced healthcare infrastructure facilitate the early detection and management of GDM [91, 92]. In contrast, LMICs bear a disproportionate burden of adverse pregnancy outcomes from GDM due to limited healthcare access, underdiagnosis and inadequate prenatal care services [93].

Diet modifications in some settings can reduce the risk of GDM, although ethnicity has been shown to moderate beneficial effects (RR 0.75 [95% CI 0.60, 0.95]) [94]. In a meta-analysis of the prevalence of GDM (*n*=71,286), a healthy diet was associated with a reduction in GDM risk among European women (OR 0.76 [95% CI 0.64, 0.90]) but not Asian, Australian or Mediterranean women (0.91 [0.78, 1.07]). Conversely, an unhealthy diet was associated with an increased risk of GDM among European (1.59 [1.41, 1.81]), Iranian (2.12 [1.12, 4.01]) and Mediterranean (1.69 [1.21, 2.23]) women but not Asian women (1.04 [0.72, 1.51]). No ethnic differences were found in the effects of diets that were meat-based or high in protein, or in the effects of diet exposure based on a dominant macronutrient (% total energy): animal protein, vegetable protein, fat or carbohydrate. A high-fish diet was associated with a lower GDM risk in European women but not Asian women [95]. Interestingly a meta-analysis of lifestyle (diet and physical activity) interventions to manage gestational weight gain reported a reduction in GDM incidence among Asian women but not European women [96].

Several studies show that South-East and East Asian women are more likely than the majority of other ethnic groups to be diagnosed as having GDM based on OGTT 1 or 2 h values, with other ethnic groups more likely to be diagnosed based on fasting glucose levels [60], which raises the question whether there are genetic differences in glucose tolerance and predisposition to GDM. In the UK, South Asian women were more likely to have a greater burden than European women of insulin deficiency and lipodystrophy multi-ancestry partitioned polygenic scores (pPSs), which in turn were most strongly associated with GDM and incident type 2 diabetes after GDM [97]. The Pacific-specific *CREBRF* rs373863828 allele has been shown to protect Mãori and Pasǐfika women against developing GDM [98]; the *HKDC1* gene confers GDM susceptibility in a South Indian population [99]; and several gene variants (including rs62069863 in *TRPV3*) are associated with increased GDM susceptibility in Chinese Han women [100]. Finally, novel genetic and molecular risk factors for GDM and glycaemic traits associated with key loci and inflammatory pathways have been shown in pregnant Chinese women [101].

## Role of geography in risk and outcomes

Seasonality has been shown to have an association with prevalence of GDM in some [102, 103] but not all populations [104]. In southern Europe, Brazil, Australia, the UK, Taiwan and Canada, post-load blood glucose values and incidence of GDM generally increase at higher ambient temperatures [102]. Even in the temperate climate of the UK, the incidence of GDM has been shown to vary by 30% from the peak incidence (October births) to the lowest incidence (March births) [105]. In colder climates, reduced physical activity during the winter months may impact the prevalence of GDM [103, 106–108]. Of importance, seasonality has been shown to be associated with some birth outcomes. The highest mean birthweight and greatest risk of emergency Caesarean delivery occur in women delivering during the spring months in the UK [105]. Meanwhile, tropical regions often face a distinct set of challenges including low rates of disease detection and limited access to preventive and therapeutic measures [109].

Pollution has also been shown to be associated with risk of GDM [110]. In one study in Los Angeles, both pre-conception and early pregnancy were reported to be susceptible windows of air pollutant exposure associated with increased GDM risk [111].

Rurality influences the risks of GDM through limited access to healthcare (delaying GDM diagnosis and management), long travel distances to healthcare facilities, limited availability of specialists, socioeconomic challenges and increased likelihood of comorbid conditions such as hypertension and obesity [112, 113]. These factors combined can increase the likelihood of complications for both mothers and neonates, emphasising the need for targeted healthcare interventions in rural settings [114]. While rurality is associated with disadvantaged care, including screening and diagnosis, urbanisation is associated with an increased risk of GDM in India [115, 116], Thailand [117], the USA [113], Turkey [118] and Tanzania [119], probably through a range of social, lifestyle and biological determinants [120].

## Conclusions

The global burden of hyperglycaemia in pregnancy is substantial, with major geographical and ethnic variations in prevalence and associated complications. Further research to better delineate the biological and social determinants of inter-ethnic disparities and outcomes is warranted. A definitive optimal approach to the detection and diagnosis of GDM in both early and later pregnancy has yet to be established. While alternatives to the OGTT have been sought and rigorously validated, it should be possible to develop a standardised approach to GDM screening and diagnosis that sits within a common framework.

Meanwhile, there is a pressing need for locally adapted, culturally appropriate prevention strategies that address the underlying determinants of health and improvements in universal access to best practice care for hyperglycaemia in pregnancy. National lifestyle programmes with local tailoring, including women with prior GDM, can be cost saving [121] and are urgently needed to reduce the impact of hyperglycaemia in pregnancy on current and future mothers.

## Supplementary Information

Below is the link to the electronic supplementary material.

Slideset of figures (PPTX 574 KB)

## Abbreviations

aOR: Adjusted OR
DIP: Diabetes in pregnancy
C&C: Carpenter and Coustan
GDM: Gestational diabetes mellitus
GCT: Glucose challenge test
IADPSG: International Association of Diabetes in Pregnancy Study groups
IFG: Impaired fasting glucose
IGT: Impaired glucose tolerance
LGA: Large for gestational age
LMICs: Low- and middle-income countries
NDDG: National Diabetes Data Group
SDOH: Social determinants of health

## Authors’ relationships and activities

Outside the submitted work, MH has received honoraria for lectures and consultancies from AstraZeneca, Boehringer Ingelheim, Eli Lilly and Novo Nordisk. Outside the submitted work, DS has received educational grants from Abbott, Ascensia and Boehringer Ingelheim, devices for research and clinical service development from AMSL and Abbott, research support from Hitachi and speaker fees from Novo Nordisk and Sanofi. The authors declare that there are no other relationships or activities that might bias, or be perceived to bias, their work.
