# Supplementary material for: Hyperglycaemia in pregnancy: the role of ethnicity and geography in risk and outcomes
Source: Diabetologia. 2025 Aug 4;68(11):2340–61. doi: 10.1007/s00125-025-06510-7 (PMC12534263; doi:10.1007/s00125-025-06510-7)
Supplement: Supplementary file 1 — Slideset of figures (PPTX 574 KB) [file 125_2025_6510_MOESM1_ESM.pptx]

## Slide 1
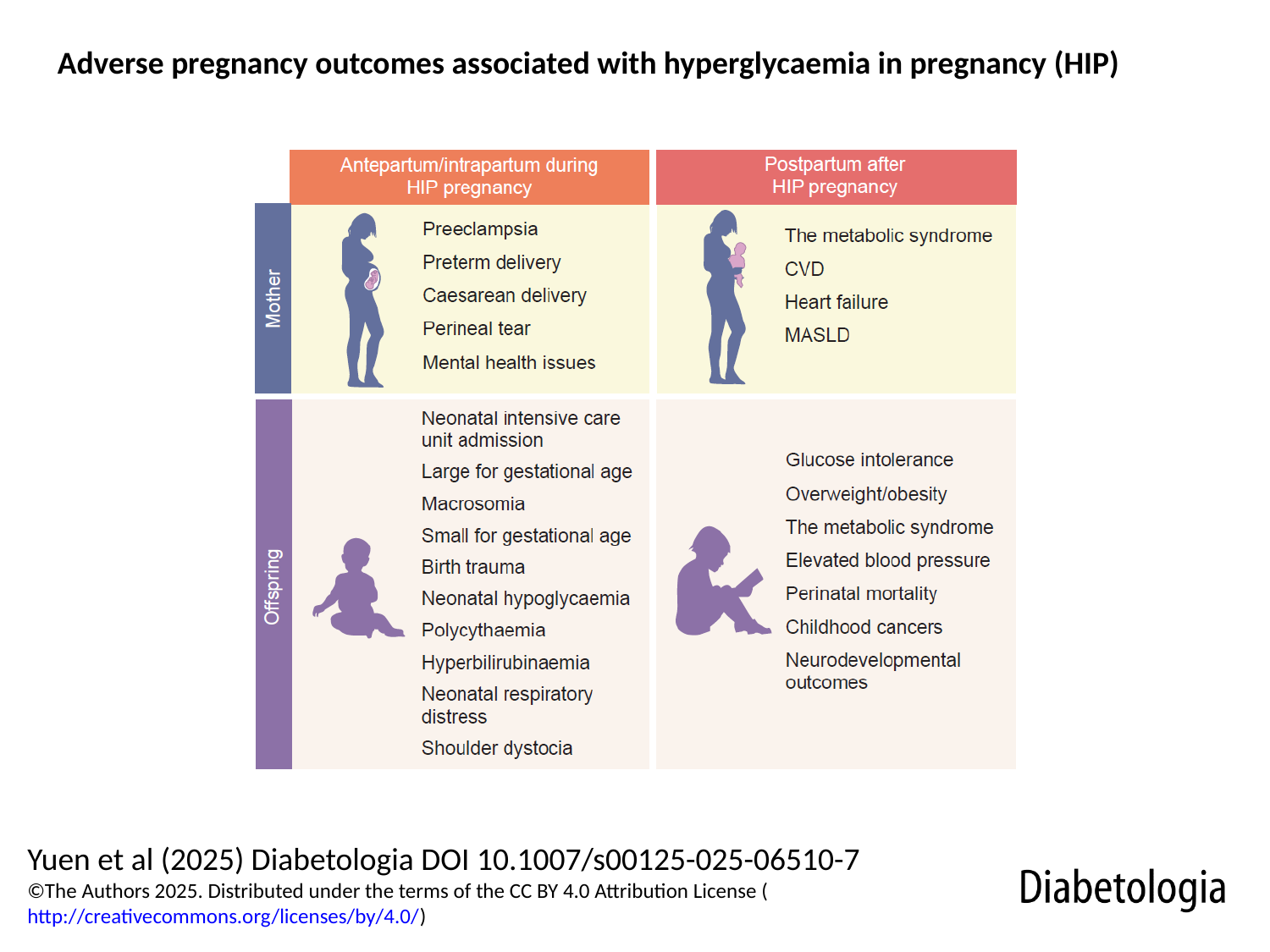

Adverse pregnancy outcomes associated with hyperglycaemia in pregnancy (HIP)
Yuen et al (2025) Diabetologia DOI 10.1007/s00125-025-06510-7
©The Authors 2025. Distributed under the terms of the CC BY 4.0 Attribution License (http://creativecommons.org/licenses/by/4.0/)

## Slide 2
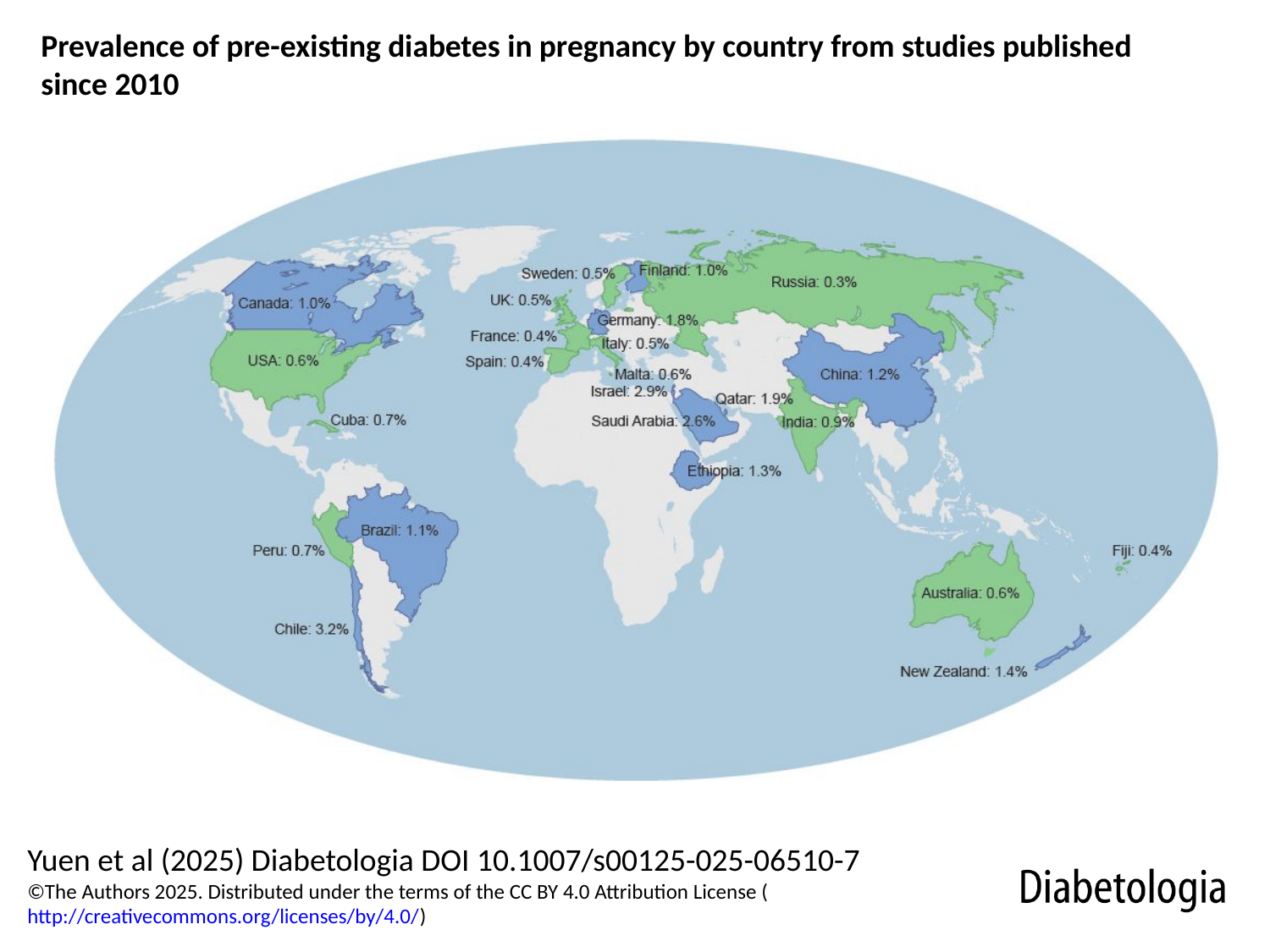

Prevalence of pre-existing diabetes in pregnancy by country from studies published
since 2010
Yuen et al (2025) Diabetologia DOI 10.1007/s00125-025-06510-7
©The Authors 2025. Distributed under the terms of the CC BY 4.0 Attribution License (http://creativecommons.org/licenses/by/4.0/)

## Slide 3
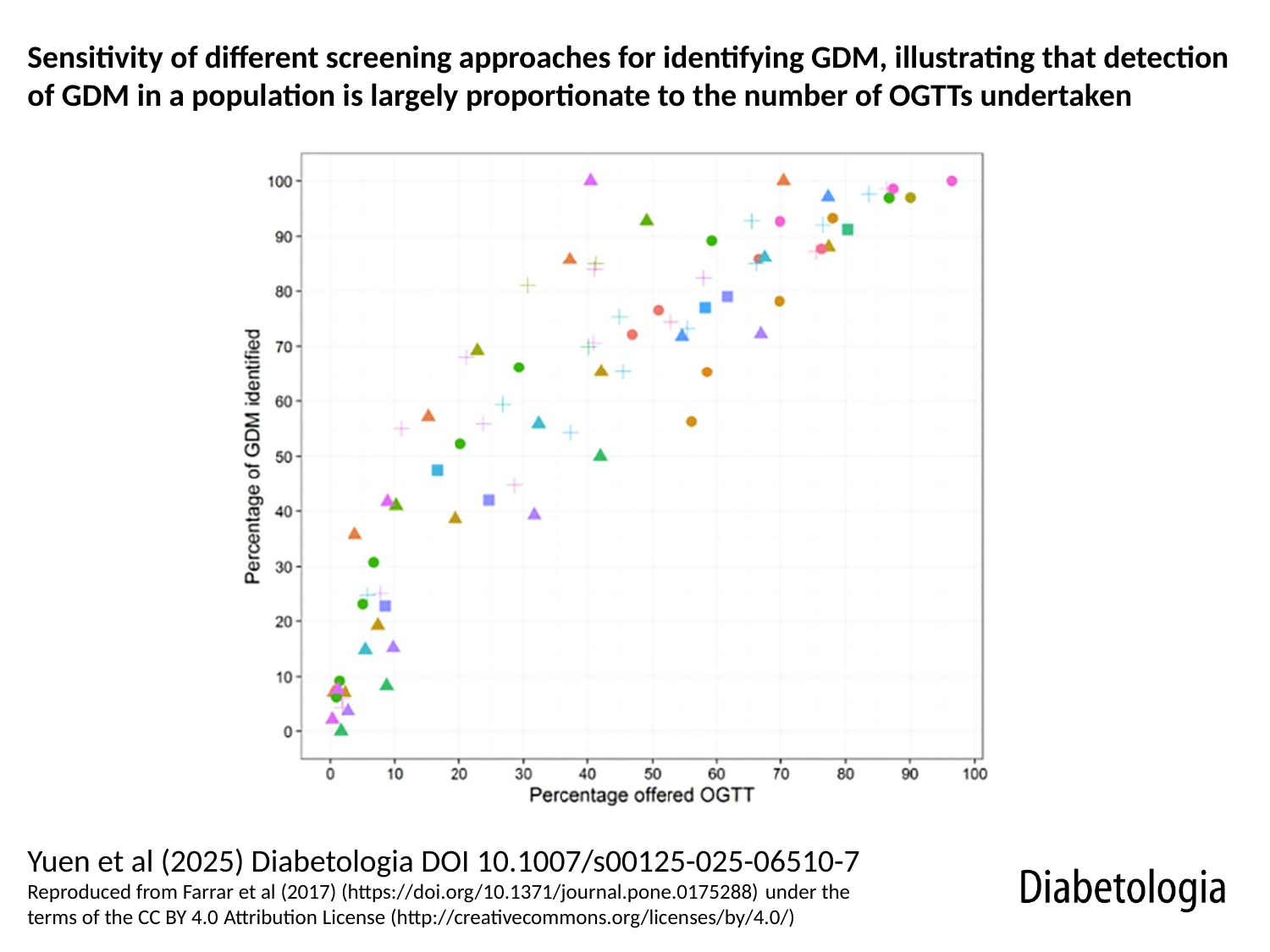

Sensitivity of different screening approaches for identifying GDM, illustrating that detection of GDM in a population is largely proportionate to the number of OGTTs undertaken
Yuen et al (2025) Diabetologia DOI 10.1007/s00125-025-06510-7
Reproduced from Farrar et al (2017) (https://doi.org/10.1371/journal.pone.0175288) under the terms of the CC BY 4.0 Attribution License (http://creativecommons.org/licenses/by/4.0/)

## Slide 4
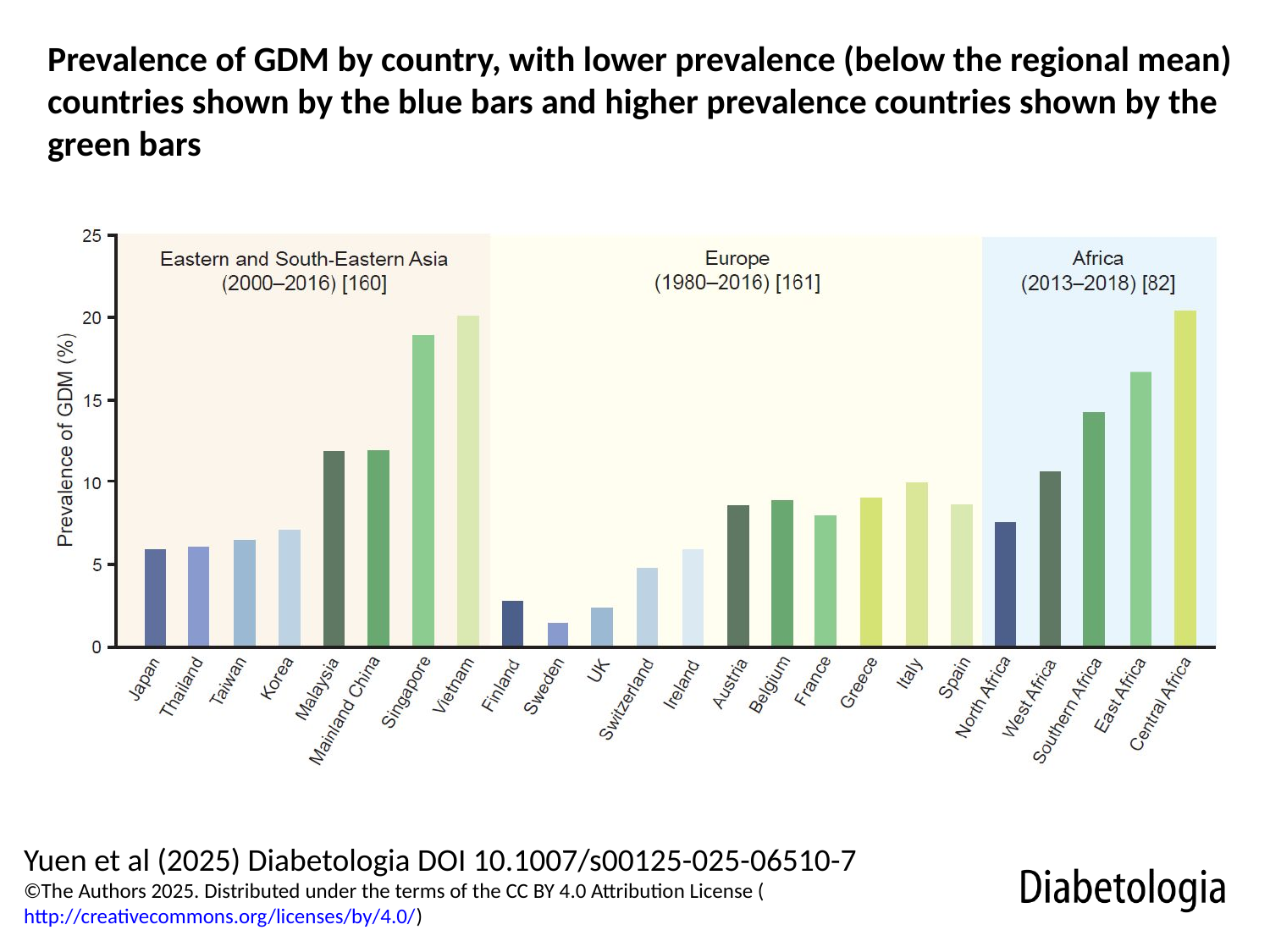

Prevalence of GDM by country, with lower prevalence (below the regional mean) countries shown by the blue bars and higher prevalence countries shown by the green bars
Yuen et al (2025) Diabetologia DOI 10.1007/s00125-025-06510-7
©The Authors 2025. Distributed under the terms of the CC BY 4.0 Attribution License (http://creativecommons.org/licenses/by/4.0/)
